# Efficacy of non-surgical periodontal treatment on patients with coronary artery disease: a meta-analysis of randomized controlled trials

**DOI:** 10.4317/medoral.25514

**Published:** 2022-10-16

**Authors:** Chundi Liu, Feifei Shi, Wenjie Li, Jun Chen

**Affiliations:** 1 Hunan Key Laboratory of Oral Health Research and Hunan 3D Printing Engineering Research Center of Oral Care and Hunan Clinical Research Center of Oral Major Diseases and Oral Health and Xiangya School of Stomatology, Central South University, Changsha, China; 2 The Affiliated Hospital of Stomatology and School of Stomatology, Zhejiang University School of Medicine and Key Laboratory of Oral Biomedical Research of Zhejiang Province, Hangzhou, China; 3 The State Key Laboratory Breeding Base of Basic Science of Stomatology and Key Laboratory for Oral Biomedicine of Ministry of Education and School and Hospital of Stomatology, Wuhan University, Wuhan, China; 4 Department of Periodontology, Xiangya Stomatological Hospital, Central South University, Changsha, China

## Abstract

**Background:**

Coronary artery disease (CAD) is defined as one of the most common cardiovascular diseases (CVDs). Periodontitis is one of the risk factors for CAD.

**Material and Methods:**

PubMed, Embase and Cochrane Library databases were carefully and thoroughly retrieved until October 2021. On the basis of the inclusion and exclusion criteria, eligible articles were selected strictly to identify randomized controlled trials (RCTs). Using Cochran's Q statistic, Review Manager 5.4 and Stata 16, data were extracted, and a comprehensive analysis was carried out.

**Results:**

Six RCTs of 619 patients were included in this study, including 360 in the intervention group (IG) and 259 in the control group (CG). Meta-analysis showed significant difference for C-reactive protein (CRP) (1.20mg/L, 95% CI: 1.13 to 1.27, *p* < 0.00001) after non-surgical periodontal therapy (NSPT), but showed no significant difference for interleukin-6 (IL-6) (1.19mg/L, 95% CI: -1.03 to 3.40, *p*=0.29), flow-mediated dilation (FMD) (-1.64%, 95% CI: -4.95 to 1.67, *p*=0.33), triacylglycerol (TG) (-0.02mg/dL, 95% CI: -0.31 to 0.27, *p*=0.90), total cholesterol (TC) (0.04mg/dL, 95% CI: -0.25 to 0.33, *p*=0.90), low-density lipoprotein cholesterol (LDL-C) (0.00mg/dL, 95% CI: -0.29 to 0.29, *p*=0.99) and high-density lipoprotein cholesterol (HDL-C) (0.11mg/dL, 95% CI: -0.18 to 0.40, *p*=0.46).

**Conclusions:**

The impact of NSPT on the reduction of CRP in patients of CAD with periodontitis is significant. NSPT can be considered as an important preventive strategy for major cardiovascular events in CAD.

** Key words:**Coronary artery disease (CAD), periodontitis, non-surgical periodontal therapy (NSPT), periodontal treatment, periodontal therapy, meta-analysis.

## Introduction

Periodontitis affects periodontal supporting tissues, consisting of gums, periodontal ligaments, cementum, and alveolar bone (1). Moreover, periodontitis is quite connected with increased systemic inflammatory factors, such as C-reactive protein (CRP), interleukin-6 (IL-6), interleukin-8 (IL-8), fibrinogen and white blood cells. Periodontitis can increase the inflammatory burden of the body, leading to the deterioration of some systemic diseases, such as cardiovascular disease (CVD) (2), diabetes (3), digestive tract cancers (4) and so on.

CVD includes coronary artery disease (CAD), cerebrovascular disease, peripheral artery disease (PAD), and aortic atherosclerosis (5). CAD accounted for the largest number of CVD, which is related to many risk factors such as smoking, obesity, hypertension and diabetes (6). Atherosclerosis is the most common pathogenesis of CAD and is closely related to vascular endothelial dysfunction. It is a chronic inflammatory process of artery wall thickening and the lumen narrowing (7) caused by local lipid accumulation, in which low-density lipoprotein (LDL) plays a vital part. CAD can remain sTable for a long time, known as chronic coronary syndrome (CCS), but may turn into acute coronary syndrome (ACS) due to unsTable conditions such as plaque rupture (8).

It has been proved that there was a positive correlation between periodontitis and CAD, in which inflammatory cytokines play an important role. Periodontal vasodilatation in patients with periodontitis can promote bacteremia, resulting in endothelial cells invaded by bacteria. Microbial accumulation in subgingival plaque has shown to be associated with arterial intima thickening (9). The mouse model has also shown that aggregatibacter actinomycetemcomitans infection leads to increased atherosclerosis (10). In addition, bacteria of oral origin have been identified by PCR in atherosclerotic plaques (11).

Non-surgical periodontal therapy (NSPT) is the main treatment for periodontitis, including supragingival scaling, subgingival scaling, root planning and oral hygiene instruction (OHI). It has been reported that periodontal treatment is beneficial to the control of CVD (12), which is a generic term for a wide range of diseases (5) including CAD, stroke, congestive heart failure, and peripheral artery disease (13). Trials involving 101 CAD patients accounted for a low proportion of the total 669 included, so there is no clear conclusion about the impact of periodontal treatment on CAD. Although CAD is one of the types of CVD, it has its particular characteristics. CAD has the highest incidence of CVDs(14). In pathological physiological aspects, CAD is characterized by coronary artery atherosclerosis unique from other CVDs (15). It presents as sTable angina, unsTable angina, myocardial infarction (MI) or sudden cardiac death (SCD) (16). Considering about a third of the sudden death of CAD patients, the early prevention and treatment of CAD are more of the essence, especially before plaque rupture leads to an acute MI or SCD. Till now, there has been no review of the effect of NSPT on CAD. In the present study, a meta-analysis was conducted, including 6 randomized controlled trials (RCTs). The objective was to assess the influence of NSPT on the cardiovascular risk in CAD with periodontitis.

## Material and Methods

- Protocol and registration

The meta-analysis was carried out conforming to the Preferred Reporting Items for Systematic Reviews and Meta-Analyses (PRISMA) scale. A protocol has been registered in the PROSPERO database (No. CRD42021285588). This article followed the protocol in terms of substance.

- Eligibility criteria

The language of the articles was restricted to English. RCTs studying the effect of NSPT on patients diagnosed as CAD with periodontitis were considered for inclusion in this study and then organized by the PICO method, according to the following points:

Types of Participants: Participants over 18 years of age diagnosed as CAD with periodontitis were considered. Periodontitis was defined as the 2018 EFP/AAP periodontitis case classification, which is elucidated as “1. interdental CAL is detecTable at ≥2 non-adjacent teeth, or 2. Buccal or oral CAL ≥3 mm with pocketing >3 mm is detecTable at ≥2 teeth and the observed CAL cannot be ascribed to non-periodontal causes.” (17). CAD was defined in the latest 2019 ESC Guidelines as a pathological process of atherosclerotic plaque deposition in the epicardial arteries, whether obstructive or not. The disease can be diagnosed by a combination of symptoms, clinical investigations and various testing results (18). Patients who had received any periodontal treatment or systemic antibiotic therapy in the past 3 months, or had less than 8 teeth remaining were excluded from the review.

Types of Intervention: NSPT in the form of supra- and sub-gingival scaling, root planning combined with OHI.

Types of Control: No periodontal treatment or delayed treatment after the study's follow-up period.

Types of Outcome measures: Systemic inflammation indexes: CRP and IL-6 were defined as the main outcome variables. Endothelial function index: flow-mediated dilatation（FMD）and lipid metabolism indexes: triglyceride (TG), total cholesterol (TC), low-density lipoprotein cholesterol (LDL-C) and high-density lipoprotein cholesterol (HDL-C) were defined as secondary outcome variables.

Length of follow-up time: Studies with a follow-up period of less than 2 months were excluded.

- Searching methods

Information sources: An electronic database search was performed in PubMed (MEDLINE), EMBASE, the Cochrane Central Register of Controlled Trials (Clinical Trials) and Web of Science. A manual search of reference studies of reviewed articles was a supplement to it.

Search strategy: All publications in English as of October 1, 2021 were searched, with no restrictions on country or article type. The following search terms were applied: “periodontal treatment”, “periodontal therapy”, “Root Scaling*”, “Root planning”, “Subgingival Scaling”, “Supragingival Scaling”, “Coronary Disease*” and “Coronary Heart Disease*”.

1.The PubMed, on 01.10.2020, using the following strategy: (((((((("Periodontal Diseases/therapy"[Mesh]) OR ("Dental Scaling"[Mesh])) OR (periodontal treatment)) OR (periodontal therapy)) OR ("Root Scaling*")) OR (Root planning)) OR (Subgingival Scaling)) OR (Supragingival Scaling)) AND (((("Coronary Disease"[MeSH]) OR ("Coronary Disease*")) OR ("Coronary Heart Disease*")) OR ("coronary artery disease*"))

2.The Embase, on 01.10.2020, using the following strategy: ((periodontal treatment) OR (periodontal therapy) OR (“Root Scaling*”) OR (Root planning) OR (Subgingival Scaling) OR (Supragingival Scaling)) AND (("Coronary Disease*") OR ("Coronary Heart Disease*") OR ("coronary artery disease*"))

3.The Web of Science, on 01.10.2020, using the following strategy:

#1 (((((ALL=(periodontal treatment)) OR ALL=(periodontal therapy)) OR ALL=(“Root Scaling*”)) OR ALL=(Root Planning)) OR ALL=(Subgingival Scaling)) OR ALL=(Supragingival Scaling)

#2 ((ALL=("Coronary Disease*")) OR ALL=("Coronary Heart Disease*")) OR ALL=("coronary artery disease*")

#1 AND #2

4.The Cochrane Central, on 01.10.2020, using the following strategy: ((periodontal treatment) OR (periodontal therapy) OR (“Root Scaling*”) OR (Root planning) OR (Subgingival Scaling) OR (Supragingival Scaling)) AND (("Coronary Disease*") OR ("Coronary Heart Disease*") OR ("coronary artery disease*"))

- Study selection

During the process of selecting studies, two investigators independently conducted the eligibility assessment in a non-blind standardized manner. Disagreements between the two reviewers were resolved through discussion with the attendance of other authors.

- Data extraction

The two authors independently extracted trial information and data from the included studies. Disagreements were resolved through discussion between the two reviewers and a review of the trial information. The following data have been extracted: author, year of publication, the country where the study was conducted, study period, original inclusion criteria, original exclusion criteria, the total number of participants included in the study, the average age of participants included in the study, main outcomes and additional outcomes.

- Assessment of risk of bias in included studies

Two reviewers performed independent and duplicate risk-of-bias assessments of the included studies applying the Cochrane Risk of Bias Tool. The reviewers evaluated the risk of bias by answering 'Low risk', 'High risk' or 'unbelievable risk' to the following items: 1. Random sequence generation (selection bias); 2. Allocation concealment (selection bias); 3. Blinding of participants and personnel (performance bias); 4. Blinding of outcome assessment (detection bias); 5. Incomplete outcome data (attrition bias); 6. Selective reporting (reporting bias); 7. Other bias. Review Manager 5.4 was applied to evaluate and graph the results of these questions.

- Data synthesis

The extracted quantitative data were pooled and combined for a statistical meta-analysis. The between-group standardized mean difference (SMD) or mean difference (MD) with 95% confidence interval (Cl) were used as expressions of the effect sizes. They were assessed by applying a fixed-effects model. The results will be presented as a forest plot. Assessment of heterogeneity between study outcomes was based on Cochran's Q statistic and the l2 Index. Review Manager 5.4 and Stata 16 were used for statistical analysis.

## Results

- Search Results

**Figure 1:**
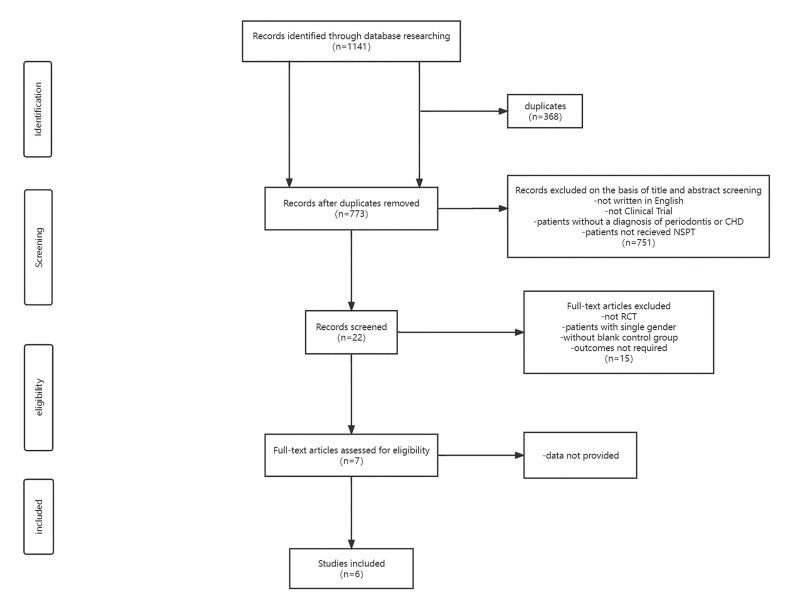
Flow chart of literature search and study selection. 6 trials were selected for analysis.

1141 records were obtained up to October 2021. 368 duplicate records were removed by endnote and manual searching. By screening the title and abstract, 751 studies were preliminarily discarded since they did not fulfill the inclusion criteria. 6 articles are included after full-text reading, and the detailed steps are presented in Fig. 1.

The overall profile of the included studies is summarized in Table 1, including study type, population characteristics, interventions, outcomes, follow-up time and so on. The studies enrolled 619 patients totally, including 360 in the intervention group (IG) and 259 in the control group (CG).

All subjects enrolled in IG received NSPT including supragingival scaling, subgingival scaling and root planning, complemented by OHI, while subjects of CG received no periodontal therapy or delayed periodontal therapy. In the studies reviewed, variables commonly used to reflect periodontal status include visible plaque, probing depth (PD), clinical attachment loss (CAL), bleeding on probing (BOP), and gingival bleeding. The main outcome measures include systemic inflammation index: CRP, IL-6, Endothelial function index: FMD, and lipid metabolism indexes: TG, TC, LDL-C, HDL-C. Most of the studies were followed up for 3-6 months, and only one study was followed up for only 2 months.

**Table 1:**
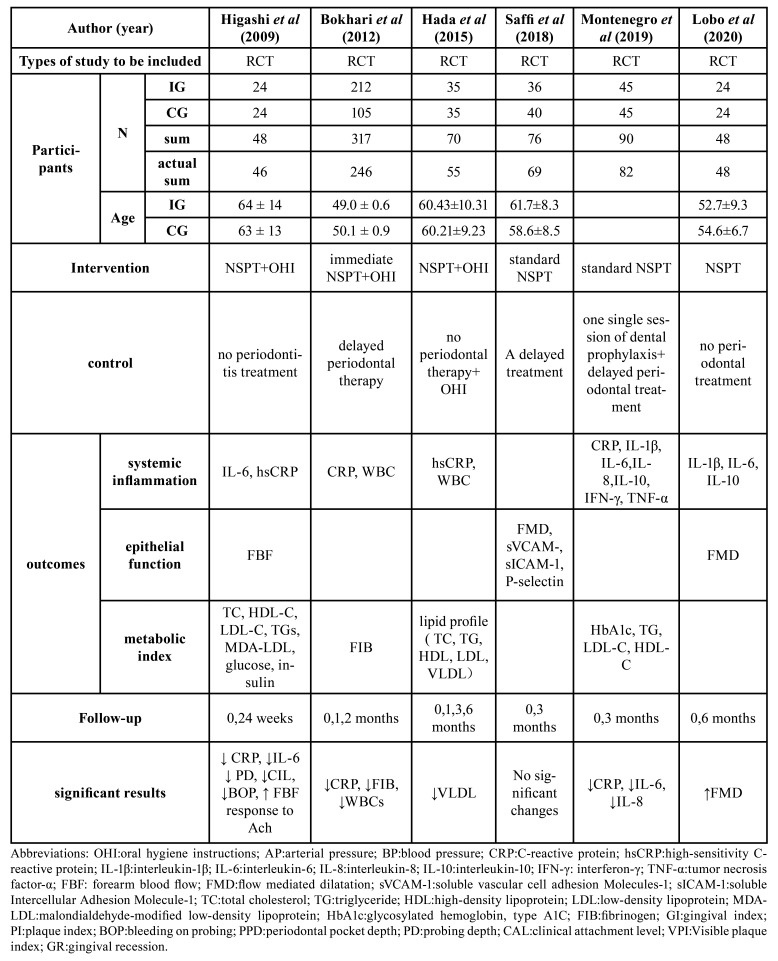
General overview of the included studies.

**Figure 2:**
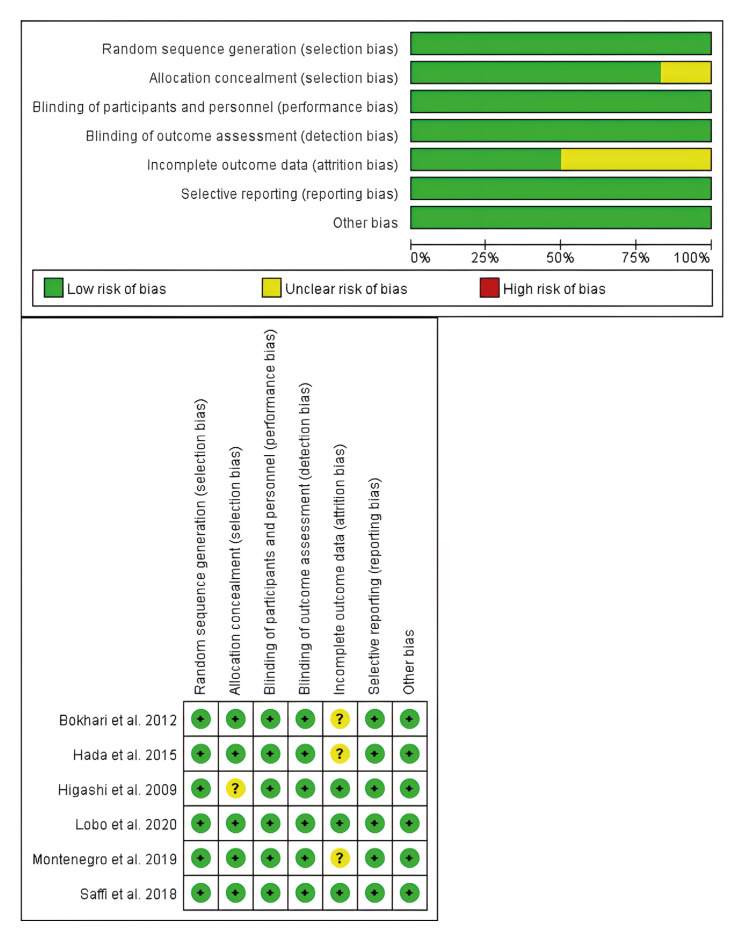
Risk of bias graph: review authors’ judgments about each risk of bias item presented as percentages across all included studies.

- Risk of bias (Fig. 2)

1. Random sequence generation: Random sequence generation method was fully described and was considered adequate.

2. Allocation concealment: The allocation sequence was random and adequately concealed.

3. Blinding of participants and personnel: Since the intervention of enrolled trials was non-surgical periodontal treatment, blinding of participants and personnel during the trial seemed impossible. It was evaluated that the results were not affected though blinding could not be achieved.

4. Blinding of outcome assessment: Blinding of outcome assessment was reported.

5. Incomplete outcome data: A small number of follow-ups were lost in the included trials. For the trials mentioned as lost to follow-up, the reasons were not clarified and the impact on inter-group balance was not clear. Therefore, the above-mentioned trials were evaluated at unclear risk owing to the lack of information available for judgment.

6. Selective reporting: All the outcomes were measured and analyzed in accordance with a pre-specified plan.

7. Other potential sources of bias: Not found.

- Results of the Meta-analysis

**Figure 3:**
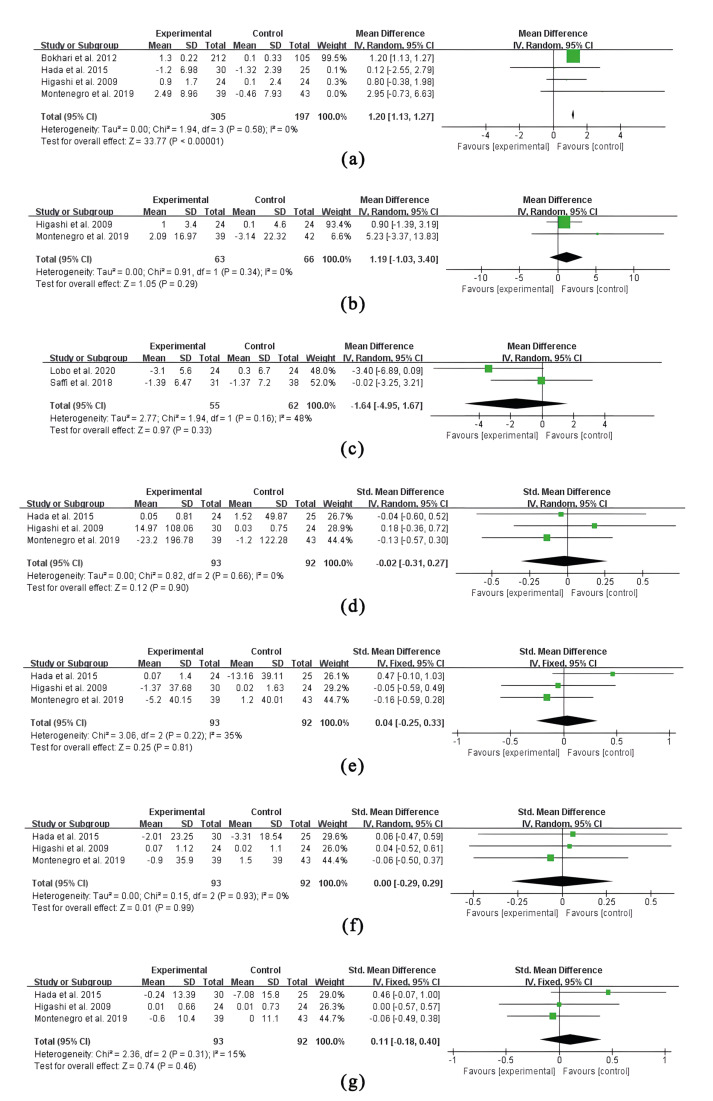
Forest plot. The meta-analysis shows the efficacy of NSPT on CRP level (a) ; IL-6 level (b) ; FMD (c) ; TG (d) ; TC (e) ; LDL-C (f) ; HDL-C (g) in periodontitis patients with CAD. SD, standard deviation; CI, confidence interval; IV, inverse variance; df, degrees of freedom.

The effect of NSPT on markers of inflammation (CRP, IL-6), vascular endothelial function (FMD) and lipid metabolism (TG, TC, LDL-C, HDL-C) have been analyzed in a series of meta-analyses. Accordingly, the mean differences of every main outcome between studies were presented in the forest plot (Fig. 3).

- CRP

(a) shows a mean difference of 1.20mg/l in CRP with a *p* value<0.00001 (95% CI: 1.13 ~ 1.27, heterogeneity I2 =0%, *P*=0.58), suggesting that NSPT significantly reduced the serum CRP level in CAD patients with periodontitis.

- IL-6

(b) shows a mean difference of 1.19mg/l in IL-6 with a *p* value=0.29 (95% CI: -1.03 to 3.40, heterogeneity I2 =0%, *P*=0.34). Compared with non-NSPT, NSPT reduced the serum level of IL-6 in CAD patients with periodontitis, but the difference was not statistically significant.

- FMD

(c) shows a mean difference of -1.64% in FMD with a *p* value=0.33 (95% CI: -4.95 to 1.67, heterogeneity I2 =48%, *P*=0.16), indicating that the increase in FMD after NSPT was not statistically significant against control. Nonetheless, it is worth mentioning that the 95%CI of the mean difference in FMD calculated by Revman or Stata is divergent from the result provided in Lobo’s study (19). As Lobo noted, the significant difference in FMD variation between the groups was 3.4% (95%CI: 0.6 to 5.8). Instead, the statistical software based on the provided data calculated a result of 3.4% (95%CI: -6.89 to 0.09), indicating that the inter-group differences in FMD were not statistically significant. Therefore, authentic conclusions cannot be drawn because the detailed original data have not been provided.

- TG, TC, LDL-C and HDL-C

(d) shows a mean difference of -0.02mg/dL in TG with a *p* value=0.90 (95% CI: -0.31 to 0.27, heterogeneity I2 =0%, *P*=0.66). (e) shows a mean difference of 0.04mg/dL in TC with a *p* value=0.90 (95% CI: -0.25 to 0.33, heterogeneity I2 =35%, *P*=0.81). (f) shows a mean difference of 0.00mg/dL in LDL-C with a *p* value=0.99 (95% CI: -0.29 to 0.29, heterogeneity I2 =0%, *P*=0.93). (g) shows a mean difference of 0.11mg/dL in HDL-C with a *p* value=0.46 (95% CI: -0.18 to 0.40, heterogeneity I2 =15%, *P*=0.31). In comparison with control, NSPT induced the increase of TG, the reduction of TC and HDL-C, and the invariance of LDL-C. In all, none of the effects of NSPT on lipid parameters were statistically significant.

## Discussion

CVD has the highest mortality rate among non-communicable diseases in the world (20). As one of the major CVD, CAD is a type of heart disease, in the coronary arteries, whose key feature is amassing of atherosclerotic plaque (18).

Traditional danger factors for CAD include hypertension, diabetes, dyslipidemia, smoking, family history of premature CAD, lack of physical activity and age. New risk factors include coronary artery calcium (CAC) score, carotid intima-media thickness (CIMT), pro-inflammatory factors, Lp(a), homocysteine, lipoprotein-associated phospholipase A2L (p-PLA2) and HDL level (21). In addition to these, more and more researches have indicated that periodontitis can be seen as one of the independent risk factors for CAD (6).

The latest advances in the connection between periodontitis and CAD can be illustrated from three aspects including epidemiological evidence, clinical association, and basic mechanism research. Periodontitis increases the danger of CAD and adverse cardiovascular events (6) has been verified in many epidemiological studies. Also, some observational studies (22,23) indicated that clinical biomarkers of oxidative stress and endothelial dysfunction were higher in CAD patients with periodontitis than that without periodontitis. Furthermore, periodontopathogens may participate in the formation and development of atherosclerosis through several mechanisms, such as bacteremia (24), oxidative stress (25), innate immune signaling (12) and endothelial dysfunction (26), etc.

In this study, data from existing RCTs were extracted and a meta-analysis was used to assess the impact of NSPT on CAD patients with periodontitis.

The important clinical indicators of CAD include CRP, IL-6, lipid index (TG, TC, LDL-C, HDL-C), FMD, fibrinogen, TNF-α, etc. Therefore, systemic inflammation index (CRP, IL-6), endothelial function index (FMD), and lipid metabolism index (TG, TC, LDL-C, HDL-C) have served as outcome indicators in this study.

CRP, an acute plasma protein, plays a crucial role in immune response and acts as a regulator (27). CRP makes a pro-inflammatory difference to endothelial cells directly(28). CRP is also an important early marker of tissue damage. It is often used as a systemic inflammatory marker of CAD as well (29). In this study, NSPT was found to significantly decrease serum CRP levels in CAD patients with periodontitis. Previous studies have reported that NSPT can significantly decrease CRP levels in periodontitis patients with CVD (12,30-32), which is consistent with our study.

IL-6 is a vital inflammatory mediator released by activated macrophages in the intima. And it is a powerful inducer of CRP (33). One prospective study (34) showed that increased IL-6 significantly increased the risk of future CAD and played a crucial part in early atherosclerosis. In this study, the reduction of IL-6 was found after NSPT, but the reduction was not statistically significant. Previous studies have drawn different conclusions on the influence of periodontal therapy on serum IL-6 levels in periodontitis patients with CVD. Some of them claim significant reduction (12,30-32) while some report a lack of effect (31). The latter reported that periodontal treatment reduced IL-6 levels, but the result had no significance.

LDL is an indispensable component of atherosclerotic plaque formation and plays a key part in the transformation of macrophages into foam cells (35). Oxidized low-density lipoprotein (Ox-LDL) and circulating LDL-derived particles give rise to the apoptosis of vascular smooth muscle cells (VSMCs) (36). It may increase atherosclerotic plaque instability and the probability of adverse cardiovascular events. HDL is involved in the reverse transport of cholesterol, affecting the progression of atherosclerosis and protecting blood vessels (37). In this study, NSPT increased TG, decreased TC and HDL-C, and kept LDL unchanged compared to non-NSPT, but the changes in lipid metabolic indexes after NPST were not statistically significant. Previous studies involving patients with periodontitis and co-morbidity, most of the reviews presented that periodontal treatment decreased TG, TC, LDL-C and increased HDL-C, but without statistical significance (30-32), comparable to our study.

Ultrasound assessment of FMD is a noninvasive and repeaTable method for evaluating endothelial dysfunction. At present, FMD is not the gold standard for evaluating CAD. But we can’t ignore that CAD is inseparable from endothelial inflammation, and severe endothelial dysfunction is an important factor affecting adverse cardiovascular events (23). The study found that NSPT led to an increase in FMD without statistical significance. It has been suggested that although NSPT has no apparent therapeutic effect on the vascular dysfunction already occurring in CAD patients, it has a good tendency to prevent further deterioration by inhibiting the development of inflammation. This is owing to proinflammatory factors playing an important role in endothelial cell injury and apoptosis (38). Endothelial function indexes including FMD and forearm blood flow (FBF) have been reported to be significantly improved by periodontal treatment in periodontitis patients with CVD (31,32), contrary to the result of this study.

In a word, based on the current meta-analysis results, NSPT can significantly reduce serum CRP levels in patients of CAD with periodontitis. Limited evidence was obtained on the effects of NSPT on other clinical indicators of CAD. CRP is the most reliable biomarker of CAD (39). It is characterized by automatic and sensitive detection, which has the most advantages in clinical application. CRP levels can be utilized to evaluate short-term prognosis and predict long-term risk after cardiovascular events. On the other hand, the use of IL-6 as a CAD biomarker is restricted by large circadian variations and a lack of verification studies (40). This may provide a possible explanation for the results of the study.

Despite some achievements that have been made in this study, there are still some limitations. Some minor modifications were made to the protocol for this review, including changing the random-effect model of the meta-analysis to a fixed-effect model according to the size of heterogeneity. It may be justified, but these changes can still be a source of bias in the review process. In addition, due to the relatively new topic of meta-analysis, few RCT studies can be included and the literature volume is small. Moreover, there were differences in the criteria of different literature, such as whether the included patients smoked or received cardiovascular care, which led to the occurrence of heterogeneity, and subgroup analysis became an unsolved problem.

Hence, there is a great need for more RCTs to investigate the efficacy of NSPT in periodontitis patients with CAD. Further RCTs with longer-term follow-up and monitoring are recommended as necessary. In future studies, it is essential to stratify study participants according to the severity degree of periodontitis. Similarly, some confounding factors including smoking and diabetes should be carefully controlled.

## Conclusions

NSPT has a positive effect on the reduction of serum CRP level in patients of CAD with periodontitis, while limited evidence was obtained that NSPT can positively affect the variation of IL-6, FMD and lipid metabolism parameters.
